# Tris(*tert*-butyl isocyanide-κ*C*)carbonylnickel(0)

**DOI:** 10.1107/S1600536808020138

**Published:** 2008-07-05

**Authors:** Wolfgang Imhof, Helmar Görls, Kathi Halbauer

**Affiliations:** a Institut für Anorganische und Analytische Chemie, Friedrich-Schiller-Universität Jena, August-Bebel-Strasse 2, 07743 Jena, Germany

## Abstract

The title compound, [Ni(C_5_H_9_N)_3_(CO)], was prepared from Ni(CO)_4_ and a tenfold excess of *tert*-butyl isocyanide. It crystallizes with two symmetry-independent mol­ecules per asymmetric unit. The central Ni atom of each independent mol­ecule has a nearly perfect tetra­hedral coordination environment, comprising one carbon monoxide and three isocyanide ligands. The title compound is the first structurally characterized Ni^0^ compound with a mixed CO/*R*NC coordination.

## Related literature

For related literature, see: Braga *et al.* (1993 ▶); Farrugia & Evans (2005 ▶); Hahn *et al.* (2004 ▶); Ladell *et al.* (1952 ▶); Bigorgne (1963*a* ▶,*b* ▶); Dönnecke & Imhof (2003 ▶); Desiraju & Steiner (1999 ▶); Halbauer *et al.* (2006 ▶, 2007 ▶); Imhof & Halbauer (2006 ▶); Imhof, Halbauer, Dönnecke & Görls (2006 ▶); Ostuka *et al.* (1969 ▶, 1971 ▶).
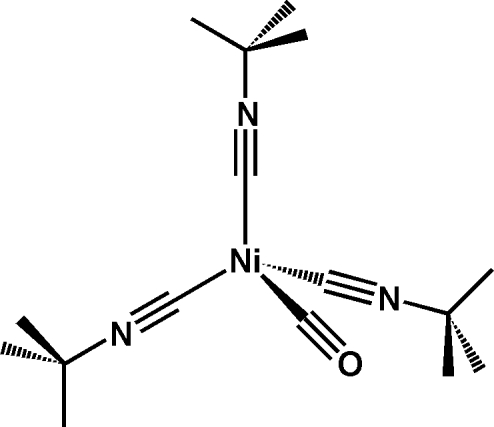


## Experimental

### 

#### Crystal data

[Ni(C_5_H_9_N)_3_(CO)]
*M* *_r_* = 336.11
Monoclinic, 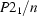

*a* = 17.1621 (7) Å
*b* = 14.5687 (5) Å
*c* = 17.1627 (7) Å
β = 113.179 (3)°
*V* = 3944.8 (3) Å^3^
*Z* = 8
Mo *K*α radiation
μ = 0.99 mm^−1^
*T* = 183 (2) K
0.06 × 0.05 × 0.05 mm

#### Data collection

Nonius KappaCCD diffractometer
Absorption correction: none
26227 measured reflections
9006 independent reflections
5006 reflections with *I* > 2σ(*I*)
*R*_int_ = 0.083

#### Refinement

*R*[*F* ^2^ > 2σ(*F* ^2^)] = 0.055
*wR*(*F* ^2^) = 0.136
*S* = 1.01
9006 reflections
397 parameters
H-atom parameters constrained
Δρ_max_ = 0.59 e Å^−3^
Δρ_min_ = −0.58 e Å^−3^

### 

Data collection: *COLLECT* (Nonius, 1998 ▶); cell refinement: *DENZO* (Otwinowski & Minor, 1997 ▶); data reduction: *DENZO*; program(s) used to solve structure: *SHELXS97* (Sheldrick, 2008 ▶); program(s) used to refine structure: *SHELXL97* (Sheldrick, 2008 ▶); molecular graphics: *XP* (Siemens, 1990 ▶); software used to prepare material for publication: *SHELXL97* and *XP*.

## Supplementary Material

Crystal structure: contains datablocks global, I. DOI: 10.1107/S1600536808020138/fj2122sup1.cif

Structure factors: contains datablocks I. DOI: 10.1107/S1600536808020138/fj2122Isup2.hkl

Additional supplementary materials:  crystallographic information; 3D view; checkCIF report

## Figures and Tables

**Fig. 1.**
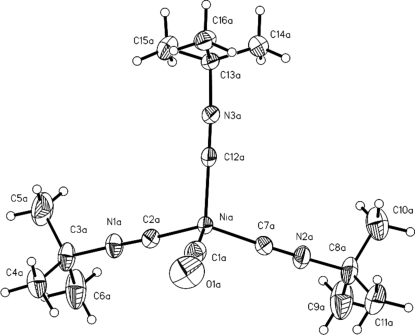
Molecular structure of one of the symmetry independent molecules of the title compound showing the labelling scheme. Displacement ellipsoids are drawn at the 40% probability level.

## supplementary crystallographic information

### Comment

Some of us recently published the synthesis of cyano complexes from the reaction of metal carbonyls with an excess of *tert*-butylisocyanide or iso-octylisocyanide, respectively (*M* = Ru: Dönnecke & Imhof (2003), Imhof & Halbauer (2006); *M* = Fe: Halbauer *et al.* (2006); *M* = Mn: Halbauer *et al.* (2007)). Mononuclear metal carbonyls like Fe(CO)_5_ or Mo(CO)_6_ under the same conditions do not react to give *M*(II) cyano complexes but yield substitution products of the corresponding carbonyl precursors (*M* = Fe: Halbauer *et al.* (2006); *M* = Mo: Imhof *et al.* (2006)). Due to the enhanced reactivity of Ni(CO)_4_ we nevertheless attempted the synthesis of complexes of the type [Ni(CN)_2_(*^t^*BuNC)_4_] from the reaction of Ni(CO)_4_ with an excess of the corresponding isocyanide leading to the formation of the title compound.

The molecular structure of one of the symmetry independent molecules of the title compound is depicted in Fig. 1. As it is expected the central nickel atom is almost perfectly tetrahedrally coordinated by three isocyanide and one carbon monoxide ligand. The metal carbon bond lengths of the isocyanide carbons atom are about 11 pm in average longer compared to the Ni—CO bond reflecting the higher π-acceptor properties of the latter. Both CO and isocyanide ligands are nearly not bent out of linearity. The bond lengths of the two molecules in the asymmetric unit are identical within experimental errors. In contrast to this observation the bond angles show slight deviations which may be caused by the bulkiness of the *tert*-butyl groups connected with packing effects. As it is expected the shortest intermolecular distances are of the C—H···O type. But whereas O1B is engaged in the three shortest interactions observed (C6A—H6AC···O1B 2.721 (8) Å; C11A—H11B···O1B 2.817 (8) Å; C16A—H16A···O1B 2.876 (8)), O1A shows only one contact below 3 Å (C16B—H16E···O1A 2.949 (9) Å). All of these contacts are well in the range discussed by Desiraju & Steiner as C—H···O hydrogen bonds (Desiraju & Steiner (1999).

With *^t^*BuNC as the ligand only [Ni(CO)_2_(*^t^*BuNC)_2_] (Ostuka *et al.* (1971)) and [Ni(*^t^*BuNC)_4_] (Ostuka *et al.* (1969)) were reported but not structurally characterized. The same is true for the compounds [Ni(CO)_4-n_(RNC)_n_] (*R* = Me, Et, *^n^*Bu, Ph; n = 1, 2, 3, 4; Bigorgne (1963a,b)). The only complexes to be structurally characterized were the homoleptic [Ni(RNC)_4_] (*R* = Ph, 2,6-Me—Ph, 2-NO_2_—Ph; Hahn *et al.* (2004)) and Ni(CO)_4_ itself (Farrugia & Evans (2005); Braga *et al.* (1993); Ladell *et al.* (1952)). So the title compound is the first [Ni(CO)_4-n_(RNC)_n_] compound to be structurally characterized.

### Experimental

0.3 ml of a 2 *M* solution of Ni(CO)_4_ (0.059 mmol) in toluene and 0.7 ml *tert*. butylisocyanide (5.86 mmol) together with another 3 ml of anhydrous toluene are transferred into a stainless steel autoclave and are heated to 130°C for 18 h. After cooling down the autoclave the resulting solution is transferrd to a Schlenk tube, all volatile material is evaporated and the resulting red oily residue is dissolved in anhydrous light petroleum (b.p. 40–60°C). After three days at -20°C the title compound crystallizes as colorless crystals. Yield: 12 mg (59%). IR (KBr pellets) [cm^-1^]: 2984*m*, 2936*m*, 2873w, 2140*m*, 2090 s, 2057 s, 2001*m*, 1920vs, 1914vs, 1453*m*, 1393*m*, 1369*m*, 1229*m*, 1208*m*. MS (DEI) [m/*z*(%)]: 336 (1) [MH^+^], 307 (13) [*M*^+^ - CO], 252 (65) [*M*^+^ - *^t^*BuNC], 224 (51) [Ni(*^t^*BuNC)_2_]^+^, 195 (10) [Ni(CO)(*^t^*BuNC)H]^+^, 168 (100) [Ni(*^t^*BuNC)(CN)H]^+^, 141 (16) [Ni(*^t^*BuNC)]^+^, 112 (99) [Ni(CO)(CN)]^+. 1^H-NMR (400 MHz, CDCl_3_, 298 K) [p.p.m.]: 1.41(*s*). ^13^C-NMR (400 MHz, CDCl_3_, 298 K) [p.p.m.]: 30.54 (CH_3_), 55.83 (C), 151.89 (NC), 197.87 (CO).

### Refinement

Hydrogen atoms were calculated in idealized positions and refined with distances of 0.96 Å. All hydrogen atoms were refined using a riding model with *U*_iso_(H) = 1.5 times *U*_iso_(C).

### Crystal data

**Table:**

[Ni(C_5_H_9_N)_3_(CO)]	*F*_000_ = 1440
*M**_r_* = 336.11	*D*_x_ = 1.132 Mg m^−^^3^
Monoclinic, *P*2_1_/*n*	Mo *K*α radiation λ = 0.71073 Å
Hall symbol: -P 2yn	Cell parameters from 26227 reflections
*a* = 17.1621 (7) Å	θ = 2.6–27.5º
*b* = 14.5687 (5) Å	µ = 0.99 mm^−^^1^
*c* = 17.1627 (7) Å	*T* = 183 (2) K
β = 113.179 (3)º	Prism, colourless
*V* = 3944.8 (3) Å^3^	0.06 × 0.05 × 0.05 mm
*Z* = 8	

### Data collection

**Table:**

Nonius KappaCCD diffractometer	5006 reflections with *I* > 2σ(*I*)
Radiation source: fine-focus sealed tube	*R*_int_ = 0.083
Monochromator: graphite	θ_max_ = 27.5º
*T* = 183(2) K	θ_min_ = 2.6º
φ and ω scans	*h* = −18→22
Absorption correction: none	*k* = −17→18
26227 measured reflections	*l* = −19→22
9006 independent reflections	

### Refinement

**Table:**

Refinement on *F*^2^	Secondary atom site location: difference Fourier map
Least-squares matrix: full	Hydrogen site location: inferred from neighbouring sites
*R*[*F*^2^ > 2σ(*F*^2^)] = 0.055	H-atom parameters constrained
*wR*(*F*^2^) = 0.136	*w* = 1/[σ^2^(*F*_o_^2^) + (0.0556*P*)^2^ + 0.2606*P*] where *P* = (*F*_o_^2^ + 2*F*_c_^2^)/3
*S* = 1.01	(Δ/σ)_max_ = 0.001
9006 reflections	Δρ_max_ = 0.59 e Å^−^^3^
397 parameters	Δρ_min_ = −0.58 e Å^−^^3^
Primary atom site location: structure-invariant direct methods	Extinction correction: none

### Special details

**Table:**

Geometry. All e.s.d.'s (except the e.s.d. in the dihedral angle between two l.s. planes) are estimated using the full covariance matrix. The cell e.s.d.'s are taken into account individually in the estimation of e.s.d.'s in distances, angles and torsion angles; correlations between e.s.d.'s in cell parameters are only used when they are defined by crystal symmetry. An approximate (isotropic) treatment of cell e.s.d.'s is used for estimating e.s.d.'s involving l.s. planes.
Refinement. Refinement of *F*^2^ against ALL reflections. The weighted *R*-factor *wR* and goodness of fit *S* are based on *F*^2^, conventional *R*-factors *R* are based on *F*, with *F* set to zero for negative *F*^2^. The threshold expression of *F*^2^ > σ(*F*^2^) is used only for calculating *R*-factors(gt) *etc*. and is not relevant to the choice of reflections for refinement. *R*-factors based on *F*^2^ are statistically about twice as large as those based on *F*, and *R*- factors based on ALL data will be even larger.

### Fractional atomic coordinates and isotropic or equivalent isotropic displacement parameters (Å^2^)

**Table:**

	*x*	*y*	*z*	*U*_iso_*/*U*_eq_	
Ni1A	0.05645 (3)	0.23726 (3)	0.89365 (3)	0.03256 (13)	
O1A	0.1424 (2)	0.3476 (2)	1.0444 (2)	0.0930 (11)	
N1A	0.15666 (18)	0.27062 (19)	0.78571 (19)	0.0442 (7)	
N2A	−0.13150 (19)	0.27889 (19)	0.8137 (2)	0.0474 (8)	
N3A	0.06472 (17)	0.03062 (18)	0.91591 (19)	0.0406 (7)	
C1A	0.1073 (3)	0.3018 (3)	0.9861 (3)	0.0495 (10)	
C2A	0.1142 (2)	0.2584 (2)	0.8233 (2)	0.0368 (8)	
C3A	0.2158 (2)	0.2906 (3)	0.7461 (2)	0.0495 (10)	
C4A	0.2853 (2)	0.3508 (3)	0.8056 (3)	0.0604 (11)	
H4AA	0.3132	0.3194	0.8600	0.091*	
H4AB	0.2608	0.4087	0.8145	0.091*	
H4AC	0.3269	0.3636	0.7810	0.091*	
C5A	0.2521 (3)	0.2004 (3)	0.7310 (3)	0.0850 (16)	
H5AA	0.2791	0.1676	0.7848	0.128*	
H5AB	0.2943	0.2128	0.7070	0.128*	
H5AC	0.2064	0.1627	0.6913	0.128*	
C6A	0.1655 (3)	0.3404 (4)	0.6631 (3)	0.0858 (16)	
H6AA	0.1387	0.3950	0.6749	0.129*	
H6AB	0.1218	0.2993	0.6252	0.129*	
H6AC	0.2038	0.3587	0.6360	0.129*	
C7A	−0.0591 (2)	0.2639 (2)	0.8422 (2)	0.0389 (8)	
C8A	−0.2222 (2)	0.2960 (3)	0.7798 (3)	0.0517 (10)	
C9A	−0.2462 (3)	0.3409 (4)	0.6944 (3)	0.0969 (18)	
H9AA	−0.2201	0.4018	0.7016	0.145*	
H9AB	−0.3080	0.3470	0.6672	0.145*	
H9AC	−0.2262	0.3030	0.6588	0.145*	
C10A	−0.2666 (3)	0.2032 (3)	0.7710 (3)	0.0737 (13)	
H10A	−0.2509	0.1635	0.7333	0.111*	
H10B	−0.3281	0.2124	0.7470	0.111*	
H10C	−0.2491	0.1743	0.8268	0.111*	
C11A	−0.2407 (3)	0.3558 (3)	0.8424 (3)	0.0732 (13)	
H11A	−0.2107	0.4144	0.8487	0.110*	
H11B	−0.2215	0.3247	0.8974	0.110*	
H11C	−0.3018	0.3672	0.8217	0.110*	
C12A	0.0622 (2)	0.1099 (2)	0.9093 (2)	0.0377 (8)	
C13A	0.0707 (2)	−0.0698 (2)	0.9185 (2)	0.0369 (8)	
C14A	−0.0192 (2)	−0.1076 (2)	0.8829 (3)	0.0506 (10)	
H14A	−0.0489	−0.0863	0.8245	0.076*	
H14B	−0.0492	−0.0861	0.9176	0.076*	
H14C	−0.0173	−0.1749	0.8838	0.076*	
C15A	0.1184 (2)	−0.0983 (2)	0.8643 (2)	0.0495 (10)	
H15A	0.0867	−0.0786	0.8057	0.074*	
H15B	0.1248	−0.1652	0.8661	0.074*	
H15C	0.1746	−0.0695	0.8862	0.074*	
C16A	0.1176 (2)	−0.0972 (2)	1.0110 (2)	0.0458 (9)	
H16A	0.1735	−0.0679	1.0337	0.069*	
H16B	0.1245	−0.1641	1.0150	0.069*	
H16C	0.0850	−0.0774	1.0437	0.069*	
Ni1B	−0.03653 (3)	0.74217 (3)	0.61221 (3)	0.03316 (13)	
O1B	−0.21523 (19)	0.7099 (2)	0.5102 (2)	0.0873 (11)	
N1B	0.06832 (18)	0.66226 (18)	0.52198 (19)	0.0408 (7)	
N2B	−0.03902 (19)	0.9488 (2)	0.62247 (19)	0.0462 (8)	
N3B	0.0221 (2)	0.64140 (19)	0.7800 (2)	0.0467 (8)	
C1B	−0.1440 (3)	0.7201 (3)	0.5515 (3)	0.0493 (10)	
C2B	0.0296 (2)	0.6961 (2)	0.5572 (2)	0.0355 (8)	
C3B	0.1153 (2)	0.6134 (2)	0.4808 (3)	0.0440 (9)	
C4B	0.1229 (6)	0.5173 (4)	0.5100 (7)	0.245 (6)	
H4BA	0.0662	0.4912	0.4951	0.368*	
H4BB	0.1540	0.5152	0.5716	0.368*	
H4BC	0.1535	0.4816	0.4826	0.368*	
C5B	0.0701 (4)	0.6220 (6)	0.3882 (4)	0.175 (4)	
H5BA	0.0166	0.5880	0.3697	0.262*	
H5BB	0.1053	0.5970	0.3601	0.262*	
H5BC	0.0582	0.6869	0.3731	0.262*	
C6B	0.2014 (3)	0.6551 (4)	0.5071 (4)	0.101 (2)	
H6BA	0.1960	0.7188	0.4874	0.152*	
H6BB	0.2346	0.6201	0.4822	0.152*	
H6BC	0.2300	0.6537	0.5690	0.152*	
C7B	−0.0316 (2)	0.8695 (2)	0.6218 (2)	0.0361 (8)	
C8B	−0.0605 (3)	1.0458 (2)	0.6121 (3)	0.0574 (11)	
C9B	0.0215 (5)	1.0972 (4)	0.6294 (5)	0.160 (4)	
H9BA	0.0617	1.0844	0.6874	0.241*	
H9BB	0.0100	1.1633	0.6229	0.241*	
H9BC	0.0457	1.0772	0.5892	0.241*	
C10B	−0.1210 (6)	1.0587 (4)	0.5234 (3)	0.190 (5)	
H10D	−0.1738	1.0260	0.5143	0.286*	
H10E	−0.0964	1.0345	0.4849	0.286*	
H10F	−0.1330	1.1243	0.5123	0.286*	
C11B	−0.0955 (3)	1.0733 (3)	0.6757 (3)	0.0795 (15)	
H11D	−0.1445	1.0347	0.6689	0.119*	
H11E	−0.1131	1.1378	0.6669	0.119*	
H11F	−0.0518	1.0655	0.7329	0.119*	
C12B	0.0011 (2)	0.6833 (2)	0.7177 (3)	0.0423 (9)	
C13B	0.0452 (2)	0.5740 (2)	0.8480 (2)	0.0468 (10)	
C14B	0.1115 (3)	0.5123 (3)	0.8381 (4)	0.0912 (18)	
H14D	0.0882	0.4831	0.7821	0.137*	
H14E	0.1281	0.4650	0.8822	0.137*	
H14F	0.1613	0.5490	0.8435	0.137*	
C15B	0.0784 (4)	0.6238 (3)	0.9314 (3)	0.0905 (18)	
H15D	0.1295	0.6581	0.9373	0.136*	
H15E	0.0921	0.5793	0.9778	0.136*	
H15F	0.0351	0.6665	0.9335	0.136*	
C16B	−0.0329 (3)	0.5185 (3)	0.8384 (3)	0.0729 (13)	
H16D	−0.0524	0.4844	0.7849	0.109*	
H16E	−0.0779	0.5600	0.8383	0.109*	
H16F	−0.0189	0.4753	0.8858	0.109*	

### Atomic displacement parameters (Å^2^)

**Table:**

	*U*^11^	*U*^22^	*U*^33^	*U*^12^	*U*^13^	*U*^23^
Ni1A	0.0291 (2)	0.0315 (2)	0.0424 (3)	0.00252 (17)	0.0198 (2)	0.00508 (19)
O1A	0.107 (3)	0.100 (3)	0.059 (2)	−0.005 (2)	0.017 (2)	−0.029 (2)
N1A	0.0384 (17)	0.0591 (18)	0.0423 (19)	0.0001 (14)	0.0235 (16)	0.0023 (14)
N2A	0.0310 (18)	0.0544 (19)	0.059 (2)	0.0067 (14)	0.0195 (16)	0.0065 (15)
N3A	0.0365 (17)	0.0355 (17)	0.055 (2)	0.0067 (12)	0.0233 (15)	0.0101 (13)
C1A	0.054 (3)	0.052 (2)	0.049 (3)	0.0049 (18)	0.027 (2)	0.001 (2)
C2A	0.0321 (18)	0.0364 (18)	0.042 (2)	0.0037 (14)	0.0146 (16)	0.0029 (15)
C3A	0.037 (2)	0.084 (3)	0.036 (2)	0.0044 (19)	0.0229 (19)	0.004 (2)
C4A	0.044 (3)	0.087 (3)	0.056 (3)	−0.008 (2)	0.025 (2)	0.004 (2)
C5A	0.073 (3)	0.107 (4)	0.101 (4)	0.001 (3)	0.062 (3)	−0.023 (3)
C6A	0.046 (3)	0.167 (5)	0.049 (3)	0.006 (3)	0.024 (2)	0.028 (3)
C7A	0.038 (2)	0.0364 (18)	0.048 (2)	0.0006 (15)	0.0237 (19)	0.0067 (16)
C8A	0.029 (2)	0.080 (3)	0.047 (3)	0.0111 (18)	0.0161 (19)	0.016 (2)
C9A	0.054 (3)	0.165 (5)	0.074 (4)	0.024 (3)	0.027 (3)	0.052 (4)
C10A	0.046 (3)	0.108 (4)	0.062 (3)	−0.013 (2)	0.016 (2)	0.001 (3)
C11A	0.055 (3)	0.090 (3)	0.087 (4)	0.013 (2)	0.041 (3)	−0.005 (3)
C12A	0.0289 (19)	0.040 (2)	0.050 (3)	0.0031 (14)	0.0220 (18)	0.0062 (16)
C13A	0.038 (2)	0.0294 (17)	0.048 (2)	0.0064 (14)	0.0219 (18)	0.0076 (15)
C14A	0.041 (2)	0.043 (2)	0.068 (3)	−0.0038 (16)	0.023 (2)	−0.0006 (18)
C15A	0.046 (2)	0.059 (2)	0.048 (3)	0.0130 (18)	0.023 (2)	0.0020 (18)
C16A	0.053 (2)	0.042 (2)	0.046 (3)	0.0088 (16)	0.024 (2)	0.0057 (17)
Ni1B	0.0321 (2)	0.0330 (2)	0.0378 (3)	−0.00067 (17)	0.0174 (2)	−0.00341 (19)
O1B	0.0411 (19)	0.140 (3)	0.085 (3)	−0.0278 (18)	0.0283 (18)	−0.053 (2)
N1B	0.0389 (17)	0.0412 (16)	0.049 (2)	0.0042 (13)	0.0247 (16)	−0.0002 (14)
N2B	0.052 (2)	0.0366 (17)	0.044 (2)	0.0012 (13)	0.0119 (16)	−0.0018 (13)
N3B	0.059 (2)	0.0396 (17)	0.047 (2)	0.0062 (14)	0.0271 (18)	0.0058 (15)
C1B	0.043 (2)	0.060 (2)	0.053 (3)	−0.0105 (18)	0.027 (2)	−0.0198 (19)
C2B	0.0324 (19)	0.0351 (18)	0.038 (2)	−0.0013 (14)	0.0129 (17)	0.0000 (15)
C3B	0.041 (2)	0.043 (2)	0.058 (3)	0.0052 (15)	0.030 (2)	−0.0093 (18)
C4B	0.374 (13)	0.045 (3)	0.525 (18)	0.053 (5)	0.401 (14)	0.044 (6)
C5B	0.056 (4)	0.388 (13)	0.071 (5)	0.054 (5)	0.014 (3)	−0.090 (6)
C6B	0.045 (3)	0.172 (5)	0.094 (4)	−0.019 (3)	0.037 (3)	−0.065 (4)
C7B	0.034 (2)	0.043 (2)	0.032 (2)	0.0000 (15)	0.0128 (17)	−0.0001 (15)
C8B	0.096 (3)	0.0279 (19)	0.053 (3)	0.0079 (19)	0.034 (3)	0.0045 (17)
C9B	0.202 (8)	0.051 (3)	0.311 (12)	−0.035 (4)	0.189 (8)	−0.033 (5)
C10B	0.348 (12)	0.101 (4)	0.046 (4)	0.130 (6)	−0.005 (5)	0.005 (3)
C11B	0.123 (4)	0.054 (3)	0.077 (4)	0.022 (3)	0.056 (3)	0.008 (2)
C12B	0.049 (2)	0.0350 (19)	0.052 (3)	−0.0004 (15)	0.029 (2)	−0.0063 (18)
C13B	0.066 (3)	0.0312 (19)	0.048 (3)	0.0040 (17)	0.028 (2)	0.0081 (17)
C14B	0.104 (4)	0.064 (3)	0.132 (5)	0.031 (3)	0.074 (4)	0.039 (3)
C15B	0.147 (5)	0.059 (3)	0.044 (3)	−0.022 (3)	0.015 (3)	0.004 (2)
C16B	0.090 (4)	0.056 (3)	0.076 (4)	−0.013 (2)	0.037 (3)	0.007 (2)

### Geometric parameters (Å, °)

**Table:**

Ni1A—C1A	1.753 (4)	Ni1B—C1B	1.755 (4)
Ni1A—C2A	1.864 (3)	Ni1B—C7B	1.861 (3)
Ni1A—C7A	1.867 (4)	Ni1B—C2B	1.864 (3)
Ni1A—C12A	1.872 (3)	Ni1B—C12B	1.874 (4)
O1A—C1A	1.155 (5)	O1B—C1B	1.156 (4)
N1A—C2A	1.162 (4)	N1B—C2B	1.169 (4)
N1A—C3A	1.457 (4)	N1B—C3B	1.451 (4)
N2A—C7A	1.162 (4)	N2B—C7B	1.163 (4)
N2A—C8A	1.453 (4)	N2B—C8B	1.454 (4)
N3A—C12A	1.159 (4)	N3B—C12B	1.158 (4)
N3A—C13A	1.466 (4)	N3B—C13B	1.457 (5)
C3A—C4A	1.509 (5)	C3B—C5B	1.475 (7)
C3A—C5A	1.519 (6)	C3B—C4B	1.475 (6)
C3A—C6A	1.526 (5)	C3B—C6B	1.493 (5)
C4A—H4AA	0.9800	C4B—H4BA	0.9800
C4A—H4AB	0.9800	C4B—H4BB	0.9800
C4A—H4AC	0.9800	C4B—H4BC	0.9800
C5A—H5AA	0.9800	C5B—H5BA	0.9800
C5A—H5AB	0.9800	C5B—H5BB	0.9800
C5A—H5AC	0.9800	C5B—H5BC	0.9800
C6A—H6AA	0.9800	C6B—H6BA	0.9800
C6A—H6AB	0.9800	C6B—H6BB	0.9800
C6A—H6AC	0.9800	C6B—H6BC	0.9800
C8A—C9A	1.507 (6)	C8B—C10B	1.479 (7)
C8A—C11A	1.511 (5)	C8B—C11B	1.492 (5)
C8A—C10A	1.529 (6)	C8B—C9B	1.515 (7)
C9A—H9AA	0.9800	C9B—H9BA	0.9800
C9A—H9AB	0.9800	C9B—H9BB	0.9800
C9A—H9AC	0.9800	C9B—H9BC	0.9800
C10A—H10A	0.9800	C10B—H10D	0.9800
C10A—H10B	0.9800	C10B—H10E	0.9800
C10A—H10C	0.9800	C10B—H10F	0.9800
C11A—H11A	0.9800	C11B—H11D	0.9800
C11A—H11B	0.9800	C11B—H11E	0.9800
C11A—H11C	0.9800	C11B—H11F	0.9800
C13A—C15A	1.519 (4)	C13B—C15B	1.503 (6)
C13A—C16A	1.523 (5)	C13B—C16B	1.517 (5)
C13A—C14A	1.522 (5)	C13B—C14B	1.512 (5)
C14A—H14A	0.9800	C14B—H14D	0.9800
C14A—H14B	0.9800	C14B—H14E	0.9800
C14A—H14C	0.9800	C14B—H14F	0.9800
C15A—H15A	0.9800	C15B—H15D	0.9800
C15A—H15B	0.9800	C15B—H15E	0.9800
C15A—H15C	0.9800	C15B—H15F	0.9800
C16A—H16A	0.9800	C16B—H16D	0.9800
C16A—H16B	0.9800	C16B—H16E	0.9800
C16A—H16C	0.9800	C16B—H16F	0.9800
			
C1A—Ni1A—C2A	107.16 (16)	C1B—Ni1B—C7B	103.66 (16)
C1A—Ni1A—C7A	111.99 (16)	C1B—Ni1B—C2B	109.91 (15)
C2A—Ni1A—C7A	113.31 (15)	C7B—Ni1B—C2B	112.86 (14)
C1A—Ni1A—C12A	114.96 (17)	C1B—Ni1B—C12B	111.80 (17)
C2A—Ni1A—C12A	104.26 (13)	C7B—Ni1B—C12B	112.61 (14)
C7A—Ni1A—C12A	105.07 (14)	C2B—Ni1B—C12B	106.11 (14)
C2A—N1A—C3A	174.3 (4)	C2B—N1B—C3B	175.5 (3)
C7A—N2A—C8A	178.4 (4)	C7B—N2B—C8B	171.2 (4)
C12A—N3A—C13A	175.1 (3)	C12B—N3B—C13B	169.3 (4)
O1A—C1A—Ni1A	176.1 (4)	O1B—C1B—Ni1B	176.7 (4)
N1A—C2A—Ni1A	174.0 (3)	N1B—C2B—Ni1B	175.9 (3)
N1A—C3A—C4A	108.1 (3)	N1B—C3B—C5B	109.0 (3)
N1A—C3A—C5A	108.4 (3)	N1B—C3B—C4B	107.0 (3)
C4A—C3A—C5A	110.5 (3)	C5B—C3B—C4B	112.7 (6)
N1A—C3A—C6A	106.7 (3)	N1B—C3B—C6B	109.0 (3)
C4A—C3A—C6A	111.2 (4)	C5B—C3B—C6B	109.3 (4)
C5A—C3A—C6A	111.8 (4)	C4B—C3B—C6B	109.8 (5)
C3A—C4A—H4AA	109.5	C3B—C4B—H4BA	109.5
C3A—C4A—H4AB	109.5	C3B—C4B—H4BB	109.5
H4AA—C4A—H4AB	109.5	H4BA—C4B—H4BB	109.5
C3A—C4A—H4AC	109.5	C3B—C4B—H4BC	109.5
H4AA—C4A—H4AC	109.5	H4BA—C4B—H4BC	109.5
H4AB—C4A—H4AC	109.5	H4BB—C4B—H4BC	109.5
C3A—C5A—H5AA	109.5	C3B—C5B—H5BA	109.5
C3A—C5A—H5AB	109.5	C3B—C5B—H5BB	109.5
H5AA—C5A—H5AB	109.5	H5BA—C5B—H5BB	109.5
C3A—C5A—H5AC	109.5	C3B—C5B—H5BC	109.5
H5AA—C5A—H5AC	109.5	H5BA—C5B—H5BC	109.5
H5AB—C5A—H5AC	109.5	H5BB—C5B—H5BC	109.5
C3A—C6A—H6AA	109.5	C3B—C6B—H6BA	109.5
C3A—C6A—H6AB	109.5	C3B—C6B—H6BB	109.5
H6AA—C6A—H6AB	109.5	H6BA—C6B—H6BB	109.5
C3A—C6A—H6AC	109.5	C3B—C6B—H6BC	109.5
H6AA—C6A—H6AC	109.5	H6BA—C6B—H6BC	109.5
H6AB—C6A—H6AC	109.5	H6BB—C6B—H6BC	109.5
N2A—C7A—Ni1A	176.7 (3)	N2B—C7B—Ni1B	171.8 (3)
N2A—C8A—C9A	107.6 (3)	N2B—C8B—C10B	106.9 (3)
N2A—C8A—C11A	107.8 (3)	N2B—C8B—C11B	109.1 (3)
C9A—C8A—C11A	112.8 (4)	C10B—C8B—C11B	113.3 (5)
N2A—C8A—C10A	107.5 (3)	N2B—C8B—C9B	106.6 (4)
C9A—C8A—C10A	110.6 (4)	C10B—C8B—C9B	111.2 (5)
C11A—C8A—C10A	110.2 (3)	C11B—C8B—C9B	109.5 (4)
C8A—C9A—H9AA	109.5	C8B—C9B—H9BA	109.5
C8A—C9A—H9AB	109.5	C8B—C9B—H9BB	109.5
H9AA—C9A—H9AB	109.5	H9BA—C9B—H9BB	109.5
C8A—C9A—H9AC	109.5	C8B—C9B—H9BC	109.5
H9AA—C9A—H9AC	109.5	H9BA—C9B—H9BC	109.5
H9AB—C9A—H9AC	109.5	H9BB—C9B—H9BC	109.5
C8A—C10A—H10A	109.5	C8B—C10B—H10D	109.5
C8A—C10A—H10B	109.5	C8B—C10B—H10E	109.5
H10A—C10A—H10B	109.5	H10D—C10B—H10E	109.5
C8A—C10A—H10C	109.5	C8B—C10B—H10F	109.5
H10A—C10A—H10C	109.5	H10D—C10B—H10F	109.5
H10B—C10A—H10C	109.5	H10E—C10B—H10F	109.5
C8A—C11A—H11A	109.5	C8B—C11B—H11D	109.5
C8A—C11A—H11B	109.5	C8B—C11B—H11E	109.5
H11A—C11A—H11B	109.5	H11D—C11B—H11E	109.5
C8A—C11A—H11C	109.5	C8B—C11B—H11F	109.5
H11A—C11A—H11C	109.5	H11D—C11B—H11F	109.5
H11B—C11A—H11C	109.5	H11E—C11B—H11F	109.5
N3A—C12A—Ni1A	177.5 (3)	N3B—C12B—Ni1B	175.4 (3)
N3A—C13A—C15A	107.6 (3)	N3B—C13B—C15B	108.6 (3)
N3A—C13A—C16A	107.2 (3)	N3B—C13B—C16B	108.8 (3)
C15A—C13A—C16A	112.0 (3)	C15B—C13B—C16B	110.4 (4)
N3A—C13A—C14A	107.5 (3)	N3B—C13B—C14B	106.8 (3)
C15A—C13A—C14A	111.2 (3)	C15B—C13B—C14B	112.0 (4)
C16A—C13A—C14A	111.1 (3)	C16B—C13B—C14B	110.1 (3)
C13A—C14A—H14A	109.5	C13B—C14B—H14D	109.5
C13A—C14A—H14B	109.5	C13B—C14B—H14E	109.5
H14A—C14A—H14B	109.5	H14D—C14B—H14E	109.5
C13A—C14A—H14C	109.5	C13B—C14B—H14F	109.5
H14A—C14A—H14C	109.5	H14D—C14B—H14F	109.5
H14B—C14A—H14C	109.5	H14E—C14B—H14F	109.5
C13A—C15A—H15A	109.5	C13B—C15B—H15D	109.5
C13A—C15A—H15B	109.5	C13B—C15B—H15E	109.5
H15A—C15A—H15B	109.5	H15D—C15B—H15E	109.5
C13A—C15A—H15C	109.5	C13B—C15B—H15F	109.5
H15A—C15A—H15C	109.5	H15D—C15B—H15F	109.5
H15B—C15A—H15C	109.5	H15E—C15B—H15F	109.5
C13A—C16A—H16A	109.5	C13B—C16B—H16D	109.5
C13A—C16A—H16B	109.5	C13B—C16B—H16E	109.5
H16A—C16A—H16B	109.5	H16D—C16B—H16E	109.5
C13A—C16A—H16C	109.5	C13B—C16B—H16F	109.5
H16A—C16A—H16C	109.5	H16D—C16B—H16F	109.5
H16B—C16A—H16C	109.5	H16E—C16B—H16F	109.5

## References

[bb1] Bigorgne, M. (1963*a*). *Bull. Soc. Chim. Fr.* pp. 295–303.

[bb2] Bigorgne, M. (1963*b*). *J. Organomet. Chem.* **1**, 101–119.

[bb3] Braga, D., Grepioni, F. & Orpen, A. G. (1993). *Organometallics*, **12**, 1481–1483.

[bb4] Desiraju, G. R. & Steiner, T. (1999). *The Weak Hydrogen Bond*, IUCr Monographs on Crystallography No. 9. Oxford Science Publications.

[bb5] Dönnecke, D. & Imhof, W. (2003). *Dalton Trans.* pp. 2737–2744.

[bb6] Farrugia, L. J. & Evans, C. (2005). *J. Phys. Chem. A*, **109**, 8834–8848. 10.1021/jp053107n 16834287

[bb7] Hahn, F. E., Münder, M. & Fröhlich, P. (2004). *Z. Naturforsch. Teil B*, **59**, 850–854.

[bb8] Halbauer, K., Dönnecke, D., Görls, H. & Imhof, W. (2006). *Z. Anorg. Allg. Chem.* **632**, 1477–1482.

[bb9] Halbauer, K., Görls, H., Fidler, T. & Imhof, W. (2007). *J. Organomet. Chem.* **692**, 1898–1911.

[bb10] Imhof, W. & Halbauer, K. (2006). *Acta Cryst.* E**62**, m1514–m1516.

[bb11] Imhof, W., Halbauer, K., Dönnecke, D. & Görls, H. (2006). *Acta Cryst.* E**62**, m462–m464.

[bb12] Ladell, J., Post, B. & Fankuchen, I. (1952). *Acta Cryst.* **5**, 795–800.

[bb13] Nonius (1998). *COLLECT.* Nonius BV, Delft, The Netherlands.

[bb14] Ostuka, S., Nakamura, Y. & Yoshida, T. (1969). *J. Am. Chem. Soc.* **91**, 6994–6999.

[bb15] Ostuka, S., Yoshida, T. & Tatsuno, Y. (1971). *J. Am. Chem. Soc.* **93**, 6462–6469.

[bb16] Otwinowski, Z. & Minor, W. (1997). *Methods in Enzymology*, Vol. 276, *Macromolecular Crystallography*, Part A, edited by C. W. Carter Jr & R. M. Sweet, pp. 307–326. New York: Academic Press.

[bb17] Sheldrick, G. M. (2008). *Acta Cryst.* A**64**, 112–122. 10.1107/S0108767307043930 18156677

[bb18] Siemens (1990). *XP.* Siemens Analytical X-ray Instruments Inc., Madison, Wisconsin, USA.

